# Coarse Alignment Technology on Moving Base for SINS Based on the Improved Quaternion Filter Algorithm

**DOI:** 10.3390/s17061424

**Published:** 2017-06-17

**Authors:** Tao Zhang, Yongyun Zhu, Feng Zhou, Yaxiong Yan, Jinwu Tong

**Affiliations:** 1 School of Instrument Science and Engineering, Southeast University, Nanjing 210096, China; zhyy@seu.edu.cn (Y.Z.); 220132619@seu.edu.cn (F.Z.); 220162808@seu.edu.cn (Y.Y.); 230139522@seu.edu.cn (J.T.); 2 Key Laboratory of Micro-Inertial Instrument and Advanced Navigation Technology, Ministry of Education, Nanjing 210096, China

**Keywords:** strapdown inertial navigation system (SINS), coarse alignment, improved quaternion filter algorithm, Doppler velocity log (DVL)

## Abstract

Initial alignment of the strapdown inertial navigation system (SINS) is intended to determine the initial attitude matrix in a short time with certain accuracy. The alignment accuracy of the quaternion filter algorithm is remarkable, but the convergence rate is slow. To solve this problem, this paper proposes an improved quaternion filter algorithm for faster initial alignment based on the error model of the quaternion filter algorithm. The improved quaternion filter algorithm constructs the K matrix based on the principle of optimal quaternion algorithm, and rebuilds the measurement model by containing acceleration and velocity errors to make the convergence rate faster. A doppler velocity log (DVL) provides the reference velocity for the improved quaternion filter alignment algorithm. In order to demonstrate the performance of the improved quaternion filter algorithm in the field, a turntable experiment and a vehicle test are carried out. The results of the experiments show that the convergence rate of the proposed improved quaternion filter is faster than that of the tradition quaternion filter algorithm. In addition, the improved quaternion filter algorithm also demonstrates advantages in terms of correctness, effectiveness, and practicability.

## 1. Introduction

The strapdown inertial navigation system (SINS) plays an important role in both military and civil navigation fields, and has become a core navigation system because of advantages in autonomy, continuity, and comprehensiveness [1]. Inertial sensors and the Doppler velocity log are always treated as primary navigation devices of vehicles [2]. SINS can track the position and orientation of the carrier relative to a known starting point based on the measurements provided by the self-contained accelerometers and gyroscopes [3,4]. The process of roughly estimating the initial attitude transformation matrix of the carrier is called coarse alignment [5].

In-motion coarse alignment is performed using a swing base or a moving base. Since the carrier has no linear velocity using a swing base, other external auxiliary information is not required to complete the process of coarse alignment. Currently, typical coarse alignment algorithms in the inertial frame on a swing base include three categories: a solidified analytical algorithm based on double-vector attitude determination, an optimal quaternion algorithm solving the Wahba [6] problem, and a filter algorithm for parameter estimation. Gaiffe proposed a coarse alignment algorithm on the swing base by analyzing the influence of the carrier movement and gravitational acceleration on initial alignment [7,8,9]. Yan presented an initial alignment method with anti-rocking disturbance based on a frequency domain isolation operator [10], which restrained linear disturbance when using a swing case. In recent years, many scholars have treated the initial alignment as a process of Wahba [6] attitude determination or parameter estimation of the filter algorithms. Li proposed a new alignment algorithm for shipborne SINS based on an in-movement filter quaternion estimation [11], which had higher alignment accuracy and faster convergence rate. Gao proposed a quaternion estimation algorithm, which was better, in term of convergence speed and accuracy, than the attitude determination algorithm based on a double vector method [12]. Wang insisted that linear vibration was an essential factor affecting the accuracy of initial alignment, so he proposed a method to restrain the linear vibration noise which used a low-pass filter to handle the projection of specific force in the inertial coordinate [13].

The research on in-motion initial alignment on a moving base mainly includes two main methods: one is transfer alignment based on a master inertial navigation system, the other is integrated alignment aided by magnetic compass, odometer, global positioning system (GPS), Doppler velocity log (DVL) or other external information. Integrated alignment on a moving base is generally completed by an integrated filter algorithm based on a SINS error model and external auxiliary information. The SINS error model mainly includes the Ψ angle error model and the Φ angle error model [14]. In 1990s, Goshen-Meskin and Bar-Itzhack proposed an observability analysis theory based on a piece-wise constant system, which provided the theoretical foundation for initial alignment on a moving base [15,16]. Alignment methods on a moving base mainly include an algorithm in the inertial coordinate and a filtering algorithm based on modern estimation theory. Xu analyzed the impact of linear movement on the compass algorithm, and introduced an in-motion compass alignment algorithm with the assistance of external reference velocity [17]. Silson proposed a rapid coarse alignment algorithm on the moving base, assisted by velocity and GPS location information [18]. Wang studied in-motion initial alignment using the Kalman filter method assisted by an odometer, the corresponding vehicle experiment showed that the alignment accuracy was less than 0.1° [19]. Wang considered in-motion alignment as a problem of Wahba [6] attitude determination by updating the quaternion using the velocity of DVL [20]. 

In this paper, the alignment error of in-motion coarse alignment is analyzed, and the problem of slow convergence for the quaternion filter algorithm has been solved. An improved quaternion filter algorithm, based on Kalman filter, is proposed; it not only improves the accuracy and convergence rate of coarse alignment using a swing base, but also completes coarse alignment on a moving base. The performance of this proposed method is better than that of the optimal quaternion algorithm and quaternion filter algorithm. Finally, the results of the experiments based on a turntable and a vehicle confirm the effectiveness and stability of the proposed algorithm.

## 2. Traditional Coarse Alignment Based on Inertial System

### 2.1. Optimal Quaternion Algorithm

The initial alignment of SINS determines attitudes by vector observations. The method of using solidification coordinate based on double vectors for attitude determination uses two vectors at different time to seek initial attitude matrix. The optimal quaternion algorithm updates the attitude matrix in the inertial coordinate to reflect the real-time actual attitude changes of the carriers, making full use of all vector information.

If the coordinate is treated as a rigid body, it is easy to describe the angle and position information of the coordinate using the quaternion. According to the quaternion arithmetic theory [12,13],

(1) $$q_{b}^{n}(t)=q_{i_{b0}}^{n}(t)q_{b}^{i_{b0}}(t)=q_{i_{b0}}^{n}(t_{0},t)⊗q_{i_{b0}}^{n}(t_{0})⊗q_{b}^{i_{b0}}(t_{0},t)⊗q_{b}^{i_{b0}}(t_{0}).$$

At the initial moment $t_{0}$, as the coordinate *i_b0_* is coincident with the coordinate *b*, $q_{i}^{b}(t_{0})={\left[\begin{array}{cccc} 1 & 0 & 0 & 0 \end{array}\right]}^{T}$, and Equation (1) can be simplified as:

(2) $$\begin{array}{ll} q_{b}^{n}(t) & =q_{i_{b0}}^{n}(t_{0},t)⊗q_{b}^{n}(t_{0})⊗q_{i_{b0}}^{b}(t_{0})⊗q_{b}^{i_{b0}}(t_{0},t)⊗q_{b}^{i_{b0}}(t_{0})\\ & =q_{i_{b0}}^{n}(t_{0},t)⊗q_{b}^{n}(t_{0})⊗q_{b}^{i_{b0}}(t_{0},t) \end{array}$$

where, $q_{b}^{n}(t_{0})$ is the attitude quaternion from body (*b*) coordinate to the navigation (*n*) coordinate at $t_{0}$, $q_{i_{b0}}^{n}(t_{0},t)$ and $q_{b}^{i_{b0}}(t_{0},t)$ are separately the changes of $q_{i_{b0}}^{n}$ and $q_{b}^{i_{b0}}$ from $t_{0}$ to t. The update of $q_{i_{b0}}^{n}(t_{0},t)$ and $q_{b}^{i_{b0}}(t_{0},t)$ can be calculated using differential equations:

(3) $$\{\begin{array}{c} \left\{ \begin{array}{l} {\dot{q}}_{b}^{i_{b0}}(t_{0},t)=\frac{1}{2}q_{b}^{i_{b0}}(t_{0},t)⊗\omega_{ib}^{b}\\ q_{b}^{i_{b0}}(t_{0},t_{0})={\left[\begin{array}{cccc} 1 & 0 & 0 & 0 \end{array}\right]}^{T} \end{array}\right.\\ \left\{ \begin{array}{l} {\dot{q}}_{n}^{i_{b0}}(t_{0},t)=\frac{1}{2}q_{n}^{i_{b0}}(t_{0},t)⊗\omega_{in}^{n}\\ q_{n}^{i_{b0}}(t_{0},t_{0})={\left[\begin{array}{cccc} 1 & 0 & 0 & 0 \end{array}\right]}^{T} \end{array}\right. \end{array}$$

where, $q_{n}^{i_{b0}}(t_{0},t)$ is the conjugate quaternion of $q_{i_{b0}}^{n}(t_{0},t)$.

For simplicity, Equation (1) can be described as

(4) $$q_{b}^{n}=q_{i_{b0}}^{n}⊗q_{b_{0}}^{n_{0}}⊗q_{b}^{i_{b0}}.$$

From Equation (4), it is clear that $q_{i_{b0}}^{n}$ and $q_{b}^{i_{b0}}$ can be calculated using Equation (3). Therefore, for calculating $q_{b}^{n}$, it is key to obtain the constant quaternion $q_{b_{0}}^{n_{0}}$.

In fact, specific force equation can be simplified as follows:

(5) $$g^{n}=q_{b}^{n}⊗\left[{\dot{v}}^{b}(t)+\omega_{ib}^{b}(t)\times v^{b}-f_{sf}^{b}(t)\right]⊗{q_{b}^{n}}^{*}$$

where, $g^{n}$ is the acceleration of gravity in *n* coordinate, $v^{b}$ is the theoretical value of velocity in *b* coordinate, $\omega_{ib}^{b}$ is the theoretical value of angular velocity in *b* coordinate, and $f_{sf}^{b}$ is the theoretical value of acceleration in *b* coordinate. The superscript * indicates the conjugate quaternion of the corresponding quaternion.

Equation (5) can be further transformed:

(6) $$q_{n}^{i_{b0}}⊗g^{n}⊗{q_{n}^{i_{b0}}}^{*}=q_{b_{0}}^{n_{0}}⊗\left(q_{b}^{i_{b0}}⊗[{\dot{v}}^{b}(t)+\omega_{ib}^{b}(t)\times v^{b}-f_{sf}^{b}(t)]⊗q_{b}^{{i_{b0}}^{*}}\right)⊗q_{b_{0}}^{{n_{0}}^{*}}.$$

Use integral algorithm on both sides of the equation to eliminate the interferer acceleration error.

(7) $$\int_{t_{k}}^{t_{k+1}}q_{n}^{i_{b0}}⊗g^{n}⊗{q_{n}^{i_{b0}}}^{*}dt=q_{b_{0}}^{n_{0}}⊗\int_{t_{k}}^{t_{k+1}}q_{b}^{i_{b0}}⊗[{\dot{v}}^{b}(t)+\omega_{ib}^{b}(t)\times v^{b}-f_{sf}^{b}(t)]⊗q_{b}^{{i_{b0}}^{*}}dt⊗q_{b_{0}}^{{n_{0}}^{*}}$$

where, $t_{k}$ is the *k*th calculation moment.

The variables α(t) and β(t) are defined as follows:

(8) $$\alpha \left(t\right)=\int_{t_{k}}^{t_{k+1}}q_{b}^{i_{b0}}⊗[{\dot{v}}^{b}(t)+\omega_{ib}^{b}(t)\times v^{b}-f_{sf}^{b}(t)]⊗q_{b}^{{i_{b0}}^{*}}dt$$

(9) $$\beta \left(t\right)=\int_{t_{k}}^{t_{k+1}}q_{n}^{i_{b0}}⊗g^{n}⊗{q_{n}^{i_{b0}}}^{*}dt.$$

Then

(10) $$\beta \left(t\right)=q_{b_{0}}^{n_{0}}⊗\alpha \left(t\right)⊗{q_{b_{0}}^{n_{0}}}^{*}.$$

Equation (10) can be further transformed:

(11) $$\left[M\left(\beta \left(t\right)\right)-M\left(\alpha \left(t\right)\right)\right]q_{b_{0}}^{n_{0}}=0_{4\times 1}$$

where, M(β(t)) and M(α(t)) are the matrices of four dimensions as follows.

(12) $$\begin{array}{cc} M\left(\beta \left(t\right)\right)=\left[\begin{array}{cc} 0 & -\beta^{T}\\ \beta & \left(\beta \times \right) \end{array}\right] & M\left(\alpha \left(t\right)\right)=\left[\begin{array}{cc} 0 & -\alpha^{T}\\ \alpha & -(\alpha \times ) \end{array}\right] \end{array}.$$

According to the determination method of optimal attitude, alignment problem can be transformed into a Wahba [6] attitude determination problem

(13) $$\begin{array}{l} \underset{q}{\min}\int_{t_{0}}^{t}\left\|\left[M\left(\beta \left(t\right)\right)-M\left(\alpha \left(t\right)\right)\right]q_{b_{0}}^{n_{0}}\right\|dt\\ =\underset{q}{\min}{q_{b_{0}}^{n_{0}}}^{*}\int_{t_{0}}^{t}\left({\left[M\left(\beta \left(t\right)\right)-M\left(\alpha \left(t\right)\right)\right]}^{T}\left[M\left(\beta \left(t\right)\right)-M\left(\alpha \left(t\right)\right)\right]\right)dt\left(q_{b_{0}}^{n_{0}}\right)\\ =\underset{q}{\min}{q_{b_{0}}^{n_{0}}}^{*}K\left(q_{b_{0}}^{n_{0}}\right) \end{array}$$

where, $K=\int_{t_{0}}^{t}\left({\left[M\left(\beta \left(t\right)\right)-M\left(\alpha \left(t\right)\right)\right]}^{T}\left[M\left(\beta \left(t\right)\right)-M\left(\alpha \left(t\right)\right)\right]\right)dt$, and quaternion $q_{b_{0}}^{n_{0}}$ satisfies the equation ${q_{b_{0}}^{n_{0}}}^{*}\left(q_{b_{0}}^{n_{0}}\right)=1$. The normalized eigenvector of matrix **K** corresponding to the smallest eigenvalue of **K** is the constant quaternion $q_{b_{0}}^{n_{0}}$.

### 2.2. Quaternion Filter Algorithm

The optimal quaternion algorithm uses the optimal attitude estimation method to obtain the initial attitude quaternion. The quaternion filter algorithm builds the linear measurement model to estimate quaternion directly [21]. In fact, the principles of quaternion filter algorithm and optimal quaternion algorithm are the same. The difference between them lies only in how to get the constant quaternion $q_{b_{0}}^{n_{0}}$. The optimal quaternion algorithm uses the optimal estimation method to obtain the eigenvectors corresponding to the minimum eigenvalue of **K** matrix, while quaternion filter algorithm calculates quaternion $q_{b_{0}}^{n_{0}}$ directly, using filter algorithm.

#### 2.2.1. Measurement Model

According to Equation (11), measurement model of the quaternion is:

(14) $$0_{4\times 1}=H_{k}Q_{k}$$

where, $H_{k}=M\left(\beta \left(k\right)\right)-M\left(\alpha \left(k\right)\right)$, $Q_{k}$ is the $q_{b_{0}}^{n_{0}}$ at moment of $t_{k}$.

Because the measurement is always zero, the measurement equation is also known as the pseudo-measurement model. For the measurement noise model, the accelerometer measurement α(k) contains measurement error, noise and random linear motion disturbance. When the carrier is moving, a measurement error of external velocity is also introduced. So the observation matrix $H_{k}$ contains various errors.

#### 2.2.2. State Space Model

The quaternion $q_{b_{0}}^{n_{0}}$ should be considered as a constant quaternion during the process of alignment. When $q_{b_{0}}^{n_{0}}$ is regarded as the estimated parameter, the state space model is very simple, as follows:

(15) $$Q_{k+1}=Q_{k}$$

where, $Q_{k+1}$ and $Q_{k}$ are the initial quaternion at moment of $t_{k+1}$ and $t_{k}$.

Combine the Equations (14) and (15) to build Kalman filtering equation for estimating the constant attitude quaternion $q_{b_{0}}^{n_{0}}$. $q_{b_{0}}^{n_{0}}$ is estimated by improved Kalman filter algorithm; the specific estimation process is as follows,

(16) $${\hat{Q}}_{k+1}={\hat{Q}}_{k}-K_{k}H_{k}{\hat{Q}}_{k}$$

(17) $$K_{k}=P_{k}H_{k}^{T}{\left[H_{k}P_{k}H_{k}^{T}+R_{k+1}\right]}^{-1}$$

(18) $$P_{k+1}=P_{k}-K_{k}[H_{k}P_{k}H_{k}^{T}+R_{k+1}]K_{k}^{T}$$

(19) $$R_{k+1}=R_{k}+[e_{k}^{2}-R_{k}]/(k+1)$$

(20) $$e_{k}=-H_{k}{\hat{Q}}_{k}$$

where, ${\hat{Q}}_{0}={\left[\begin{array}{cccc} 1 & 0 & 0 & 0 \end{array}\right]}^{T}$, $P_{0}=aI$ (a is a large positive number). $R_{0}=cI$ (the recommended value of *c* is 0.1).

The above algorithm uses the innovation $e_{k}$ to calculate the filter gain, so the statistical characteristics of velocity disturbances need not to be known. The filter gain is determined adaptively according to the innovation at each filtering moment, which can accelerate the convergence rate.

## 3. New Coarse Alignment Based on Improved Quaternion Filter Algorithm

### 3.1. Alignment Error Analysis of Quaternion Filter Algorithm

According to Equation (9), β(t) is the integration of the local gravitational acceleration g transformed from *n* coordinate to *i* coordinate. Because local gravitational acceleration can be exactly known, the observation vectors β(t) and M(β(t)) can be considered error free. On an in-motion base, the observation vector α(t) contains the integration of accelerometers, which include constant bias and random errors. Therefore, in Equation (14), the observation matrix $H_{k}$ also contains errors, which results in a slow convergence rate. The coarse alignment error of the quaternion filter algorithm is analyzed as follows.

The output of accelerometers with errors is ${\hat{f}}_{sf}^{b}=f_{sf}^{b}+\nabla^{b}+r^{b}$, where $\nabla^{b}$ is the constant bias of accelerometers and $r^{b}$ is the random error and disturbance. The output of gyroscopes with errors is ${\hat{\omega }}_{ib}^{b}=\omega_{ib}^{b}+\varepsilon^{b}+\chi^{b}$, where $\varepsilon^{b}$ is the constant drift of gyroscopes and $\chi^{b}$ is the random drift. The model of velocity with errors is ${\hat{v}}^{b}=v^{b}+\delta v^{b}$, where $\delta v^{b}$ is the error of velocity. According to Equation (8), the observation vector $v^{b_{0}}$ with errors at *k*th sampling moment is as follows.

(21) $$\begin{array}{ll} \hat{\alpha }\left(k\right) & =\int_{t_{k}}^{t_{k+1}}q_{b}^{i_{b0}}⊗[{\dot{\hat{v}}}^{b}(t)+{\hat{\omega }}_{ib}^{b}(t)\times {\hat{v}}^{b}-{\hat{f}}_{sf}^{b}(t)]⊗q_{b}^{{i_{b0}}^{*}}dt\\ & =\int_{t_{k}}^{t_{k+1}}q_{b}^{i_{b0}}⊗[{\dot{v}}^{b}(t)+\omega_{ib}^{b}(t)\times v^{b}-f_{sf}^{b}(t)]⊗q_{b}^{{i_{b0}}^{*}}dt\\ & +\int_{t_{k}}^{t_{k+1}}q_{b}^{i_{b0}}⊗\left[\delta {\dot{v}}^{b}(t)+\omega_{ib}^{b}(t)\times \delta v^{b}\right]⊗q_{b}^{{i_{b0}}^{*}}dt\\ & +\int_{t_{k}}^{t_{k+1}}q_{b}^{i_{b0}}⊗\left[\varepsilon^{b}\times v^{b}(t)+\chi^{b}\times v^{b}(t)\right]⊗q_{b}^{{i_{b0}}^{*}}dt\\ & -\int_{t_{k}}^{t_{k+1}}q_{b}^{i_{b0}}⊗\left[\nabla^{b}+r^{b}\right]⊗q_{b}^{{i_{b0}}^{*}}dt \end{array}.$$

Set the vectors as:

(22) $$\{\begin{array}{l} v=\int_{t_{k}}^{t_{k+1}}q_{b}^{i_{b0}}⊗\left[\delta {\dot{v}}^{b}(t)+\omega_{ib}^{b}(t)\times \delta v^{b}\right]⊗q_{b}^{{i_{b0}}^{*}}dt\\ \varepsilon =\int_{t_{k}}^{t_{k+1}}q_{b}^{i_{b0}}⊗\left[\varepsilon^{b}\times v^{b}(t)+\chi^{b}\times v^{b}(t)\right]⊗q_{b}^{{i_{b0}}^{*}}dt\\ \nabla =\int_{t_{k}}^{t_{k+1}}q_{b}^{i_{b0}}⊗\left[\nabla^{b}+r^{b}\right]⊗q_{b}^{{i_{b0}}^{*}}dt \end{array}.$$

Thus

(23) $$M\left(\hat{\alpha }\left(k\right)\right)=M\left(\alpha \left(k\right)\right)+M(v)+M(\varepsilon )-M(\nabla ).$$

The observation matrix $H_{k}$ with errors is ${\hat{H}}_{k}=M(\beta \left(k\right))-M(\hat{\alpha }\left(k\right))$, so:

(24) $${\hat{H}}_{k}Q_{k}=\left(H_{k}-M(v)-M(\varepsilon )+M(\nabla )\right)Q_{k}=\left(-M(v)-M(\varepsilon )+M(\nabla )\right)Q_{k}.$$

By moving the right part to the left, the following is obtained:

(25) $${\hat{H}}_{k}Q_{k}+M(v)Q_{k}+M(\varepsilon )Q_{k}-M(\nabla )Q_{k}=0_{4\times 1}.$$

From Equation (25), we see that the actual observation model includes constant bias and random error of accelerometer, constant and random drift of gyroscope, and a velocity error. However, these errors are all ignored in Equation (14), which may result in the slow convergence rate of quaternion filter algorithm.

**Figure 1:**
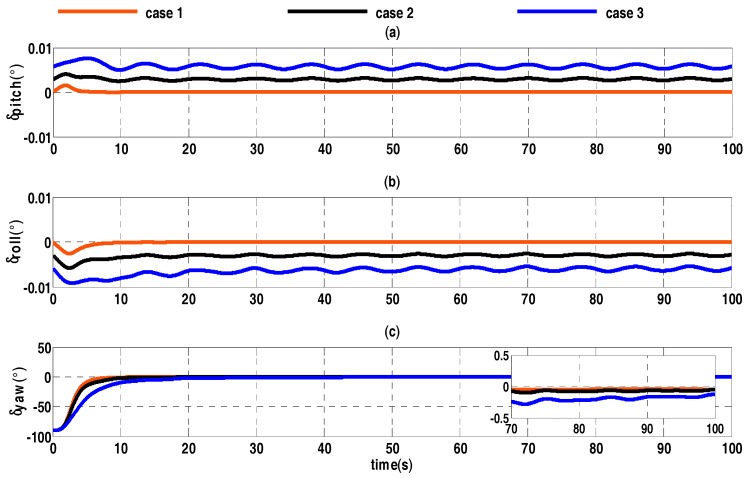
Alignment error curves of three different constant biases. (**a**) The error curves of pitch angle; (**b**) the error curves of roll angle; (**c**) the error curves of heading angle.

In order to verify the above theory, taking the constant bias of accelerometers as an example, different values of constant bias are compared in Table 1. The other simulation conditions are the same. Attitude errors of alignment are shown in Figure 1.

In Figure 1, it can be seen that the larger the constant bias, the slower the process of the coarse alignment, and lower the accuracy of alignment. In fact, accelerometer error is generally large. In addition, the constant and random drift of the gyro and velocity errors are similar to the constant bias of the accelerometer, so quaternion filter algorithm needs to be improved.

### 3.2. Improved Quaternion Filter Algorithm

As discussed above, if the constant bias and random error of accelerometer, the constant drift and random drift of gyroscope, and the velocity error can all be estimated and compensated for, the convergence rate of quaternion filter algorithm will be improved significantly. A digital filter can be designed to eliminate the disturbances of the accelerometer, gyroscope and DVL, but the design of digital filter is complicated because the characteristics of these disturbances must be analyzed, the parameters adjusted, an appropriate transitional zone designed. Yet, once the filter is designed to estimate unreasonable inputs, the useful information will also be filtered out.

According to the principles of the optimal quaternion algorithm, the quaternion filter algorithm can be improved by constructing **K** matrix to accelerate the convergence rate. K matrix is constructed as follows:

(26) $${\hat{K}}_{k}=\int_{t_{0}}^{t_{k}}{{\hat{H}}_{k}}^{T}{\hat{H}}_{k}dt=\int_{t_{0}}^{t_{k}}{\left[H_{k}-M(v)-M(\varepsilon )+M(\nabla )\right]}^{T}[H_{k}-M(v)-M(\varepsilon )+M(\nabla )]dt.$$

Ignoring the terms of the second order errors and random disturbances, Equation (26) is simplified as:

(27) $${\hat{K}}_{k}=-\int_{t_{0}}^{t_{k}}\left(H_{k}^{2}+H_{k}M(\nabla )+M(\nabla )H_{k}\right)dt+\int_{t_{0}}^{t_{k}}\left(H_{k}M(v)+M(v)H_{k}+H_{k}M(\varepsilon )+M(\varepsilon )H_{k}\right)dt.$$

Both sides of the equation are multiplied by $Q_{k}$ and according to Equation (14), then

(28) $$\begin{array}{ll} {\hat{K}}_{k}Q_{k} & =-\int_{t_{0}}^{t_{k}}\left(H_{k}^{2}+H_{k}M(\nabla )+M(\nabla )H_{k}\right)dtQ_{k}+\int_{t_{0}}^{t_{k}}\left(H_{k}M(v)+M(v)H_{k}+H_{k}M(\varepsilon )+M(\varepsilon )H_{k}\right)dtQ_{k}\\ & =\int_{t_{0}}^{t_{k}}\left[H_{k}\left(M(v)+M(\varepsilon )-M(\nabla )\right)\right]dtQ_{k}\\ & =\int_{t_{0}}^{t_{k}}\left({\hat{H}}_{k}-M(\nabla )-M(r)\right)\left(M(v)+M(\varepsilon )-M(\nabla )\right)dtQ_{k}\\ & =\int_{t_{0}}^{t_{k}}{\hat{H}}_{k}\left(M(v)+M(\varepsilon )-M(\nabla )\right)dtQ_{k} \end{array}.$$

By moving the right part to the left, the following is obtained:

(29) $${\hat{K}}_{k}Q_{k}+\int_{t_{0}}^{t_{k}}{\hat{H}}_{k}\left(M(\nabla )-M(v)-M(\varepsilon )\right)dtQ_{k}=0_{4\times 1}.$$

According to ${\hat{H}}_{k}=M(\beta \left(k\right))-M(\hat{\alpha }\left(k\right))$, it’s easy to determine that the elements of ${\hat{H}}_{k}$ are real numbers smaller than 1. Hence, the influences of accelerometer errors, gyroscope errors and velocity error in Equation (29) are greatly reduced.

If the accelerometer errors, gyroscope errors and velocity error are unknown, the observation model Equation (14) can be described by:

(30) $$0_{4\times 1}={\hat{K}}_{k}Q_{k}+v_{k}$$

where, $v_{k}$ indicates the related noises of accelerometer, gyroscope and DVL, which can be considered as the white noise with a mean of 0.

## 4. Experimental Analysis

### 4.1. Experimental Environments

In order to demonstrate the performance of the improved quaternion filter algorithm in the actual environment, two coarse alignment experiments are carried out. One is an alignment experiment on a swing base based on a three-axis turntable, while the other is an alignment experiment on a moving base based on the vehicle. The characteristics of the inertial measurement unit (IMU) used in the experiments are as follows, the constant drift stability of each gyro is less than $0.01^{\left(\circ \right)}/h\left(1\sigma \right)$, random walk coefficient is less than $0.01^{\left(\circ \right)}/h\left(1\sigma \right)$, the bias of each quartz flexible accelerometer is less than $5\times 10^{-5}g$. The initial position of the experiment is 32.05°(N) and 118.0°(E).

**Figure 2:**
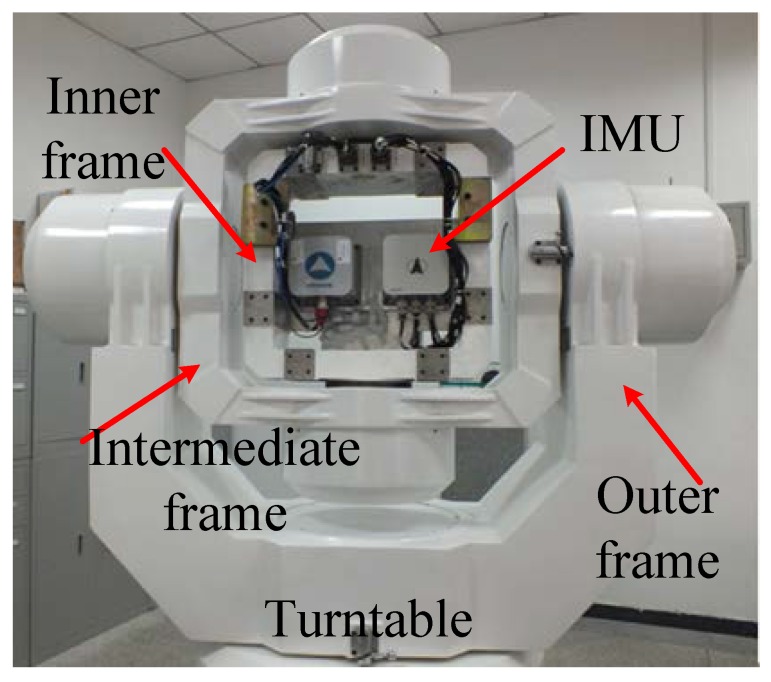
Experiment environment based on three-axis turntable.

**Figure 3:**
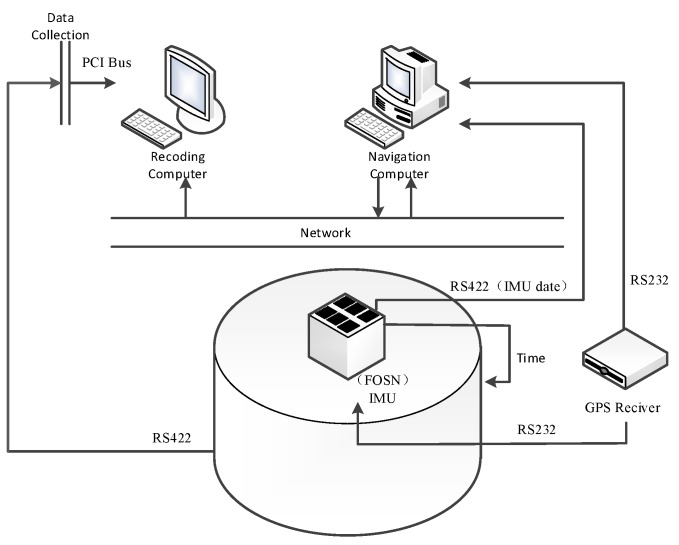
Structure diagram of experiment environment

The experiment environment on a swing base is shown in Figure 2, and the corresponding structural diagram is shown in Figure 3. It mainly consists of a fiber optic gyro inertial system (FOSN), a navigation computer (alignment algorithm is running in it), a computer used for storing, a GPS receiver, and the three-axis turntable. The navigation computer infuses outputs of the IMU and GPS receiver, and sends the result to the storing computer through the network. The storing computer compares the result of the coarse alignment from navigation computer with the attitude values of the turntable, and calculates the alignment accuracy.

**Figure 4:**
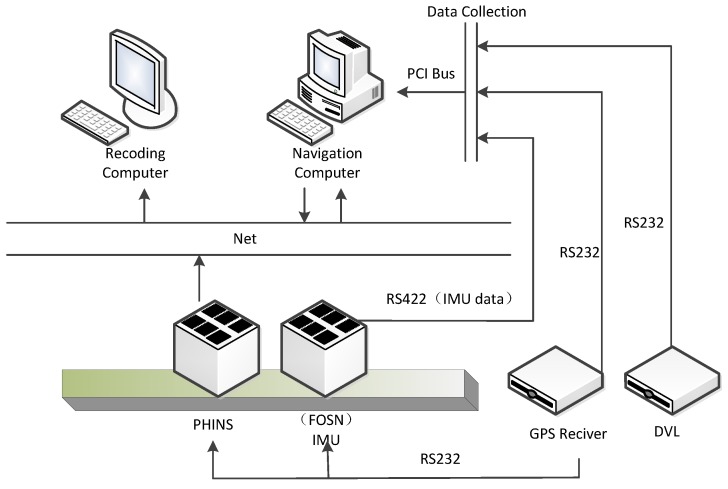
Structure diagram of alignment experiment based on vehicle.

**Figure 5:**
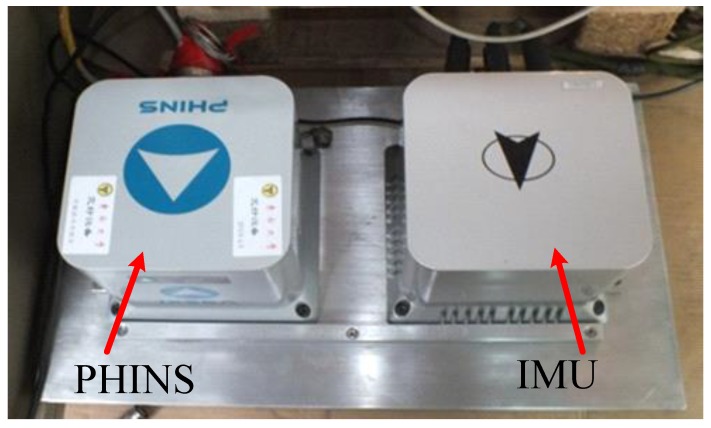
Installation method of the fiber optic gyro inertial system (FOSN) and the high-precision fiber optic gyro SINS (PHINS).

**Figure 6:**
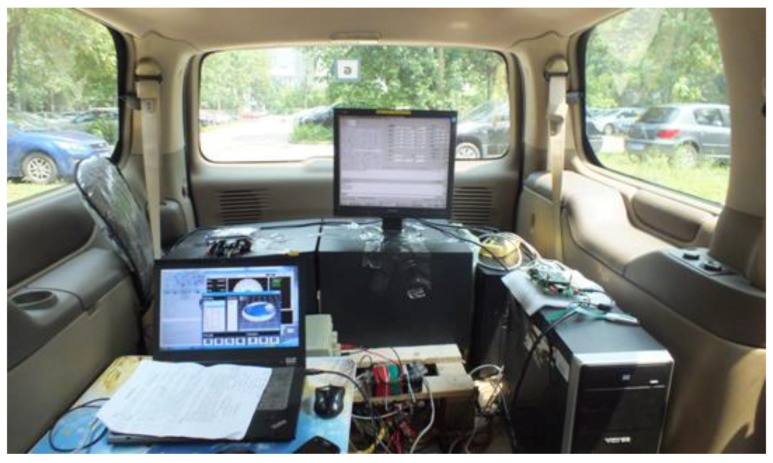
Experiment environment based on vehicle.

The structure diagram of the vehicle-based alignment experiment is shown in Figure 4. In order to evaluate the accuracy, the high-precision fiber optic gyro SINS (PHINS), produced by French company iXBlue, was chosen as a reference. The PHINS and FOSN are mounted on a rigid board with parallel heading angles, as shown in Figure 5. The experiment environment is shown in Figure 6. PHINS operates in the mode of integrated navigation aided by GPS, and sends the result to the storing computer, which is regarded as the real alignment reference. DVL provides the velocity to the navigation computer for assisting FOSN to complete the initial alignment. The storing computer stores the alignment results of FOSN and PHINS, and assesses the alignment accuracy of proposed algorithm. The project is used in the underwater environment. Since the experiment condition is limited, we use the vehicle experiment instead. The reference velocity is provided by PHINS with a constant error of 0.2 m/s and a random error of 0.005 m/s.

### 4.2. Alignment Experiment on Swing Base Based on Turntable

**Figure 7:**
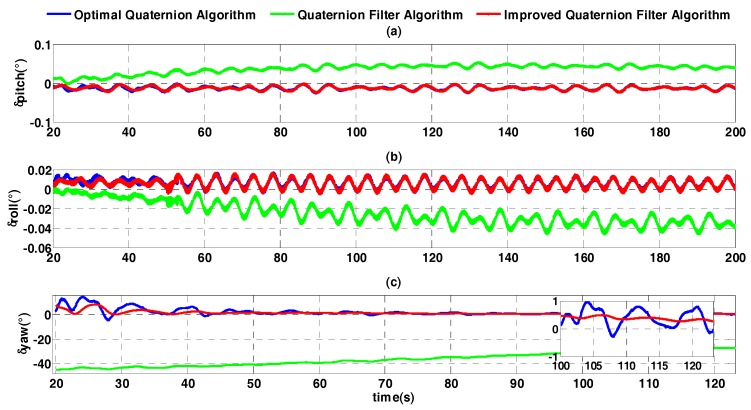
Attitude error curves of coarse alignment (the swing center of heading is 45°). (**a**) The error curves of pitch angle; (**b**) the error curves of roll angle; (**c**) the error curves of heading angle.

A FOSN is mounted on the three-axis turntable, whose swing mode is set as follows: (1) the swing center of pitch is 2°, swing amplitude is 8°, and swing frequency is 0.15 Hz; (2) the swing center of roll is −2°, swing amplitude is 10°, and swing frequency is 0.2 Hz; (3) the swing center of heading is set at multiple angles, namely 0°, 45°, 90°, 135°, 180°, 225°, 270°, and 315°, swing amplitude is 6°, and swing frequency is 0.125 Hz. To show characteristics of different algorithms, error curves of the optimal quaternion algorithm, the quaternion filter algorithm, and the improved quaternion filter algorithm are compared in Figure 7.

Figure 7 shows that the convergence rate of that quaternion filter algorithm is so slow that it is not suitable for practical use. The horizontal attitude accuracies of the optimal quaternion algorithm and the improved quaternion filter algorithm are similar to one another. The means and standard deviations of their alignment errors for heading are respectively 0.1431° and 0.1232°, and 0.1302° and 0.0315°. Although the mean values of two algorithms are close, the standard deviation of the latter is much smaller than the former because the latter is more stable.

In order to thoroughly compare the alignment effects of the optimal quaternion algorithm and the improved quaternion filter algorithm, the errors are shown at three alignment moment, such as 50 s, 80 s, and 100 s, in Table 2.

As Figure 7 shows, both optimal quaternion algorithm and improved quaternion filter algorithm can converge rapidly. However, the error curve of the heading angle of the former algorithm fluctuates at the beginning of the alignment. This is because the information on **K** matrix at the initial moment is so insufficient that the eigenvectors of **K** matrix are unstable. Although the improved quaternion filter algorithm also uses the attitude quaternion from **K** matrix, it uses an extended recursive least square algorithm, whose gain is determined adaptively, by innovation at each filtering moment. Therefore, the improved quaternion filter algorithm is more stable than the optimal quaternion algorithm. In Table 2, it can be seen that at 100 s, the alignment accuracy of the improved quaternion filter algorithm is obviously better than that of the optimal quaternion algorithm. Hence we conclude that the improved quaternion filter algorithm has distinct advantages in convergence rate and stability.

### 4.3. Alignment Experiment on Moving base Based on Vehicle

**Figure 8:**
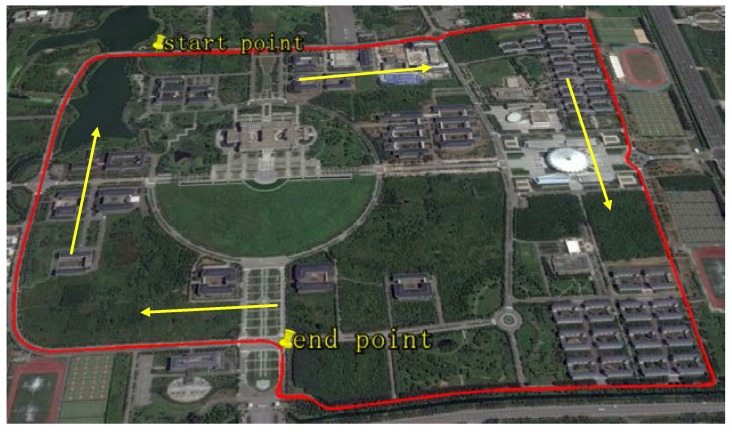
Route of the vehicle.

In order to further verify the advantages of the improved quaternion filter algorithm on the moving base assisted by external velocity, an alignment experiment based on vehicle is carried out. The route of the vehicle is shown in Figure 8, looking like a circle inside Southeast University. This experiment mainly focuses on the influence of external velocity on the alignment results.

#### 4.3.1. Influence of Filtering Frequency of External Velocity on Alignment

**Figure 9:**
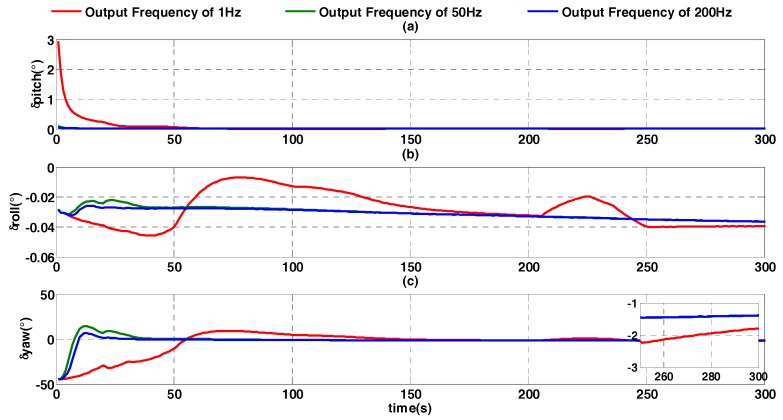
Alignment error curves at different output frequency of velocity. (**a**) The error curves of pitch angle; (**b**) the error curves of roll angle; (**c**) the error curves of heading angle.

In this experiment, DVL provides the external velocity, and its frequency is set at different value, such as 200 Hz, 50 Hz and 1 Hz. The attitude error of the improved quaternion filter algorithm is shown in Figure 9.

**Figure 10:**
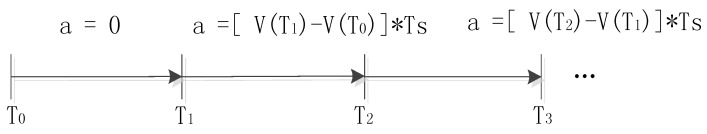
Compensation mode of velocity difference.

In Figure 9, it is shown that, at 1 Hz, the heading angle converges slowly and obviously fluctuates. With increasing velocity output frequency, the convergence rate is faster and the alignment result is better. At 300 s, the heading angle errors at 1 Hz, 50 Hz, and 200 Hz are respectively −1.788°, −1.389°, and −1.384°. In a low frequency situation such as 1Hz, the heading angle error accumulates mainly in the acceleration and deceleration process of the vehicle, because the estimation of velocity difference in every SINS calculation period is inaccurate. The compensation method is shown in Figure 10, where the velocity difference in velocity output period is evenly distributed across every strapdown calculation cycle. Ts is the SINS calculation frequency, T is the velocity output period, a is the velocity difference in SINS calculation period, and V(Ti) is the velocity value at the moment of Ti.

#### 4.3.2. Influence of Constant Error of External Velocity

**Figure 11:**
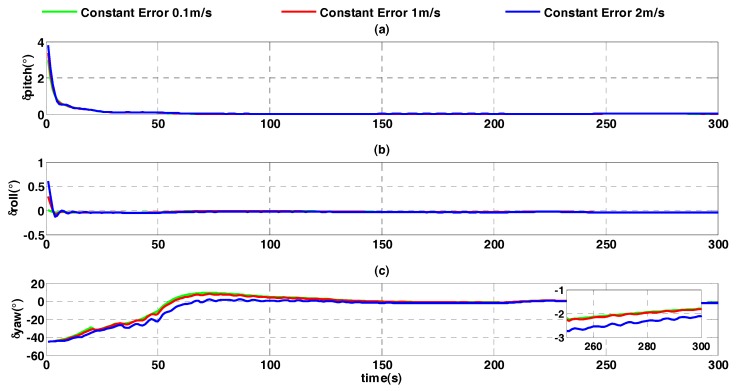
Alignment error curves of different constant errors. (**a**) The error curves of pitch angle; (**b**) the error curves of roll angle; (**c**) the error curves of heading angle.

The constant error of external velocity is set to different values, such as 2 m/s, 1 m/s and 0.1 m/s. Corresponding to different constant errors of external velocity, the alignment results of the improved quaternion filter algorithm on moving base are shown in Figure 11.

In Figure 11, it can be seen that, under conditions of different constant error of velocity, horizontal attitude error can converge fast, while the error of the heading angle increases with the increasing of constant error. The heading angle errors are similar when constant errors are 0.1m/s and 1 m/s, at which point the heading angle errors are −1.787° and −1.807°. In comparison, the heading angle error is −2.099° when constant error is 2m/s. The constant error of external reference velocity influences the alignment accuracy in accordance with the term of acceleration $\omega_{ib}^{b}\times v^{b}$, but its influence is limited.

#### 4.3.3. Influence of Random Error of External Velocity

**Figure 12:**
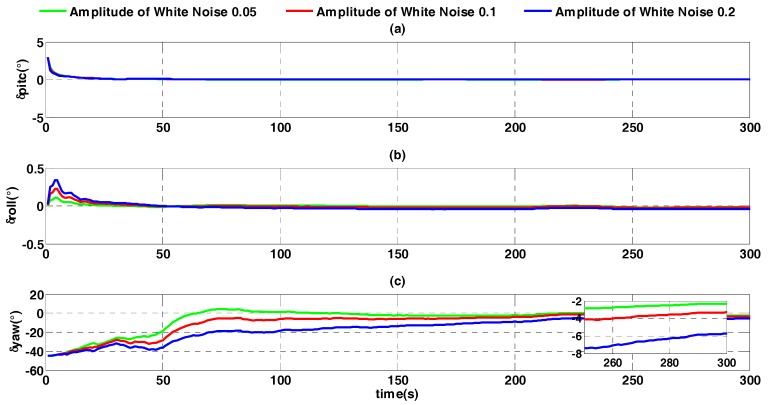
Alignment error curves with different random errors. (**a**) The error curves of pitch angle; (**b**) the error curves of roll angle; (**c**) the error curves of heading angle.

The constant error is assumed to be zero to analyze the influence of random error of velocity on the alignment accuracy. Three different values of white noise random errors are selected: 0.05 time, 0.1 time and 0.2 time. The alignment error is shown in Figure 12.

In Figure 12 it is shown that with increasing random velocity error, the alignment result converges more slowly. At the alignment moment of 300 s, heading angle errors corresponding to the random error are, from smallest to largest, −2.292°, −3.284°, and −5.722°. Compared with the influence of constant error of velocity, the influence of random error is larger. This is because random error of velocity influences the alignment accuracy by both of differential velocity ${\dot{v}}^{b}$ and $\omega_{ib}^{b}\times v^{b}$. Therefore, as shown in Figure 10 and Equation (5), during the whole process of alignment, random error has been accumulating within the external reference velocity update period.

**Figure 13:**
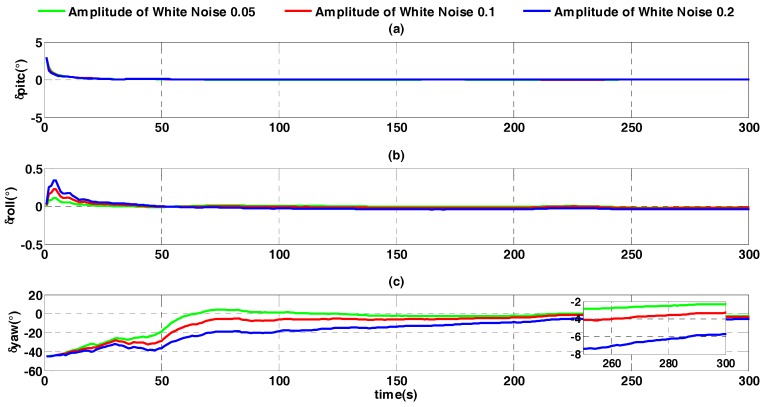
Alignment error curves of two algorithms. (**a**) The error curves of pitch angle; (**b**) the error curves of roll angle; (**c**) the error curves of heading angle.

The three experiments described above show that when the output frequency of external velocity is low, such as 1 Hz, the alignment error will increase, with external velocity becoming larger. Further, the influence of random error of external velocity on alignment result is more obvious than that of constant error. In order to compare the optimal quaternion algorithm to the improved quaternion filter algorithm described in this paper, it is more practical to assume that the velocity error model consists of constant error than random error, as in the four cases shown in the Table 3. The alignment results of the optimal quaternion algorithm and the improved quaternion filter algorithm for case 4 are as shown in Figure 13, and the heading angle errors for all case at 250 s and 500 s are shown in Table 4.

In Figure 13, it can be seen that, in the process of coarse alignment on the moving base, the optimal quaternion algorithm obviously fluctuates, while the curve of the improved quaternion filter algorithm is smooth. Therefore, the improved quaternion filter algorithm shows better stability performance. Table 4 shows that the alignment accuracies of the heading angles of the two algorithms are similar at 500 s, but the accuracy of the improved quaternion filter algorithm is better than that of the optimal quaternion algorithm at 250 s. This shows that the convergence rate of the improved quaternion filter algorithm is faster. Comparing the alignment results of case 2 and case 3 also verified that the influence of random error is larger than that of constant error.

## 5. Conclusions

Tackling the slow convergence problem of the quaternion filter algorithm, an improved quaternion filter algorithm is proposed which constructs the **K** matrix based on the principle of the optimal quaternion algorithm. Turntable- and vehicle-based experiments were carried out to verify the characteristics of the proposed algorithm. The turntable-based experimental results show that the convergence rate of the improved quaternion filter algorithm is faster than that of the quaternion filter algorithm. The vehicle-based experimental results show that the alignment accuracy of the improved quaternion filter algorithm is more stable, while the heading angle of the optimal quaternion algorithm fluctuates mightily at the beginning of alignment. In addition, the effect of the random error of external velocity on alignment accuracy is greater than that of the constant error. In summary, the improved quaternion filter algorithm can effectively improve the alignment accuracy and accelerate the convergence rate on the moving base.

## Figures and Tables

**Table 1:** Three different values of constant biases for accelerometer

Case	Constant Bias (μg)
Case 1	5
Case 2	50
Case 3	100

**Table 2:** Errors of two coarse alignment algorithms.

Algorithm	Swing Center (°) of Heading	Pitching Angle Error (°)			Rolling Angle Error (°)			Heading Angle Error (°)		
		50s	80 s	100 s	50 s	80 s	100 s	50 s	80 s	100 s
Optimal Quaternion Algorithm	0	−0.0219	−0.0056	−0.0145	0.0169	0.0171	0.0141	2.7866	0.7566	0.4148
	45	−0.0201	−0.0139	−0.0103	0.0030	0.0017	−0.0013	1.7711	1.4484	0.1193
	90	−0.0151	−0.0118	−0.0144	0.0034	0.0045	0.0037	2.7320	1.0634	0.7520
	135	−0.0113	−0.0126	−0.0185	0.0070	0.0059	0.0029	1.4054	0.3946	0.1570
	180	−0.0110	−0.0066	−0.0159	0.0087	0.0108	0.0101	1.1408	0.5920	−0.7132
	225	−0.0123	−0.0246	0.0148	0.0049	0.0050	0.0067	1.7053	0.4782	0.3951
	270	−0.0175	−0.0191	−0.0119	0.0057	0.0015	0.0042	−0.5589	−0.2711	−0.1750
	315	−0.0117	−0.0152	−0.0215	0.0100	0.0111	0.0078	0.2325	−1.4312	−0.1962
Improved Quaternion Filter Algorithm	0	−0.0178	−0.0035	−0.0137	0.0179	0.0176	0.0146	0.8022	0.1060	0.1996
	45	−0.0199	−0.0117	−0.0115	0.0026	0.0002	−0.0008	0.9761	0.5823	0.3619
	90	−0.0146	−0.0108	−0.0146	−0.0007	0.0022	0.0019	0.6502	0.3279	0.3044
	135	−0.0108	−0.0154	−0.0179	0.0064	0.0056	0.0034	0.3722	0.4190	0.3141
	180	0.0135	0.0053	−0.0034	0.0038	0.0103	0.0108	1.1376	0.5627	0.2620
	225	−0.0126	−0.0248	−0.0154	0.0041	0.0047	0.0067	1.1237	0.5357	0.3432
	270	−0.0188	−0.0198	−0.0118	0.0047	0.0018	0.0042	−0.5844	−0.3297	−0.2836
	315	−0.0091	−0.0176	−0.0208	0.0124	0.0104	0.0087	−1.2904	−0.8082	−0.4780

**Table 3:** Four cases of external reference velocity.

Case	Constant Error (m/s)	Amplitude of White Noise
Case1	0	0
Case2	1	0
Case3	0	0.1
Case4	1	0.1

**Table 4:** Heading angle errors of two algorithms (°).

Algorithm	Case 1		Case 2		Case 3		Case 4	
	250 s	500 s	250 s	500 s	250 s	500 s	250 s	500 s
Optimal Quaternion Algorithm	−3.6344	−1.1019	−3.5854	−1.1936	−5.1880	−1.3528	−5.1391	−1.3446
Improved Quaternion Filter Algorithm	−2.1934	−1.1184	−2.2696	−1.1242	−4.1223	−1.4524	−4.4928	−1.4319
